# Slippage in stacking of graphene nanofragments induced by spin polarization

**DOI:** 10.1038/srep10985

**Published:** 2015-06-16

**Authors:** Yanyu Lei, Wanrun Jiang, Xing Dai, Ruixia Song, Bo Wang, Yang Gao, Zhigang Wang

**Affiliations:** 1 Institute of Atomic and Molecular Physics, Jilin University, Changchun 130012, People’s Republic of China; 2 Jilin Provincial Key Laboratory of Applied Atomic and Molecular Spectroscopy (Jilin University), Changchun, 130012, People’s Republic of China

## Abstract

Spin polarization and stacking are interesting effects in complex molecular systems and are both presented in graphene-based materials. Their possible combination may provide a new perspective in understanding the intermolecular force. The nanoscale graphene structures with zigzag edges could possess spin-polarized ground states. However, the mechanical effect of spin polarization in stacking of graphene nanofragments is not clear. Here we demonstrate the displacement between two stacked rhombic graphene nanofragments induced by spin polarization, using first-principles density-functional methods. We found that, in stacking of two rhombic graphene nanofragments, a spin-polarized stacked conformation with zero total spin is energetically more favorable than the closed-shell stacking. The spin-polarized conformation gives a further horizontal interlayer displacement within 1 angstrom compared with the closed-shell structure. This result highlights that, besides the well-known phenomenologically interpreted van der Waals forces, a specific mechanism dependent on the monomeric spin polarization may lead to obvious mechanical effects in some intermolecular interactions.

The interactions between magnetic objects and the earth through the Earth’s magnetic field brought about the birth of compass. Analogizing this macroscopic magnetic induction, we expect to find a similar induced behavior between two molecules with a separation beyond bonding distance. So far, the magnetism of carbon-based materials has been confirmed in experiments^1^,^2^, which is attributed to the intrinsic spin polarization of carbon atoms around vacancy sites^3^,^4^. For graphene nanoribbons, the spin polarization is mainly localized at their zigzag edges^5^, and such spin-polarized ground states have been observed^6^. Other nanoscale graphene structures with zigzag edges, including several fragments and finite nanotubes, were also predicted to have spin-polarized ground states^7^,^8^,^9^,^10^ which form antiferromagnetic couplings between neighbor carbon atoms and have zero total spin (S = 0), as described by the Lieb’s theorem^11^. Based on these backgrounds and the inspiration from compass, we inferred that an intermolecular behavior response to spin polarization may be found between two spin-polarized graphene nanostructures.

The inference can be appropriately demonstrated by stacking of graphene nanofragments. Stacking is acknowledged as a typical structural balance held by intermolecular force, which is sensitive to monomeric electronic structure such as the π-electron delocalization in graphene. Realizations about this phenomenon can be traced back to the studies of structures of graphite. The concept of AB stacking for bilayer graphene originated from the Bernal structure for graphite determined in 1924^12^, and the AA stacking from the theoretical conclusions on simple hexagonal graphite^13^,^14^. Following studies on structural transition of large-scale bilayer graphene omitted the spin polarization and indicated AB stacked conformation is most stable^15^,^16^. However, spin polarization becomes non-negligible for graphene nanoribbons with zigzag edges^6^ and has been noticed in their stacking^17^,^18^. Meanwhile, infinite bilayer graphene under pressure shows interplay between monomeric electronic structure and interlayer slip^19^. Thus, two stacked graphene nanofragments may show a notable structural change between their spin-polarized and non-spin-polarized conformations, reflecting the inferred intermolecular response behavior.

According to studies and discussions above, we chose the rhombic graphene nanofragment with hydrogen passivated edge atoms to be the stacking monomer in this work, which possesses four zigzag edges and an *S* = 0 spin-polarized ground state. Then, the stacking systems of freestanding bilayer graphene nanofragments were constructed.

## Results

**Figure 1:**
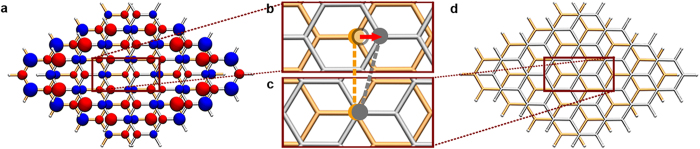
Comparison of optimized structures between the spin-polarized and the closed-shell stacked bilayer rhombic graphene nanofragments. Graphene fragments are drawn in tube models, where the orange one represents the lower fragment and the light gray one represents the upper fragment. (**a**) The top view of the obtained energetically most favorable conformation and its spin density describing an *S* = 0 spin-polarized electronic structure. The red isosurface is for the up spin and the blue isosurface is for the down spin. (**b**,**c**) Detailed stacking patterns of the spin-polarized and the closed-shell stacked conformation, respectively. To give a comparison between two stacking structures, the atoms of the lower and upper fragments are marked by orange dots and gray dots, respectively. The red arrow and the gray circle pointed out a horizontal displacement about 0.82 Å of the upper fragment in b compared with that in c. (**d**) The top view of the closed-shell stacking.

**Figure 2:**
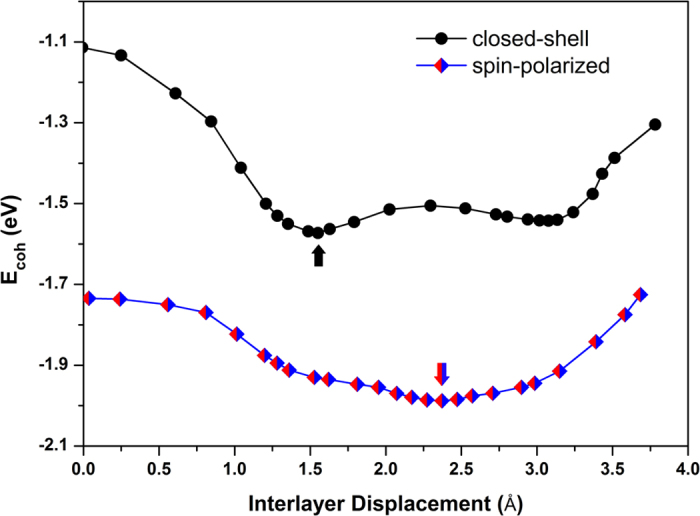
Cohesive energy curves respect to the interlayer displacement between two graphene nanofragments of closed-shell and spin-polarized conformations. The cohesive energy (*E_coh_*) is defined as the energy difference between the total energy of stacked systems and that of two free ground-state fragments (similarly defined as reference 18). The interlayer displacement varies along the slipping direction. The zero point at the horizontal axis represents the case of AA stacking. Arrows point out the closed-shell stacked conformation discussed and the optimal spin-polarized conformation, respectively.

After structural optimizations, both spin-polarized and closed-shell stacked conformations of bilayer graphene nanofragments were obtained. The ones most stable in two states respectively are shown in Fig. 1. The atomic coordinates for two conformations can be found in Supplementary Table S1 and Supplementary Table S2 online, respectively. The most stable one in spin-polarized states possess antiferromagnetic and antiferromagnetic couplings for the intra-layer and inter-layer spin arrangements, respectively (AFM-AFM). Comparison of total energy between spin-polarized conformations possessing different spin-coupling patterns can be found in Supplementary Table S3 online. We found that this *S* = 0 spin-polarized conformation has the lowest total energy which is about 0.42 eV lower than that of the closed-shell one. Furthermore, a horizontal displacement about 0.82 Å is presented between two fragments of the spin-polarized structure, compared with the closed-shell stacking. Thus, the slippage in stacking of graphene nanofragments induced by spin polarization is clearly demonstrated. The displacement is clearly shown in Fig. 2 which plots the cohesive energy curves respect to the interlayer displacement between two graphene nanofragments of conformations in closed-shell and optimal AFM-AFM spin-polarized states, respectively. This result is reproduced by another density functional method, which gave a same energetic preference for the spin-polarized conformation and a total energy lowering about 0.31 eV than that of the closed-shell stacking, with the further horizontal slippage about 0.41 Å.

In addition, the *S* =* *0 spin-polarized ground state of the single rhombic graphene nanofragment is stable when the size increases. And it’s suggested that the mechanical effect induced by spin-polarization would occur when the size of the system increases in a small range. Meanwhile, twisted stacking didn’t give a lower total energy than the optimal slipped conformation in our calculations. Comparison of the total energy among different states of the single rhombic fragment in different sizes can be found in Supplementary Table S4 online. Related information about size effect on the stacking system we studied can be found in Supplementary Table S5 online. Energy differences between twisted conformations and the optimal slipped conformation can be found in Supplementary Table S6 online.

## Discussion

Spin-polarized stacked conformations different from the AA and AB stacking have been reported in bilayer zigzag graphene nanoribbons^17^,^18^. Because their optimal conformations are nonmagnetic, the spin-polarization would not spontaneously induce the interlayer displacement in these periodic systems. In contrast, the slipped spin-polarized conformation is optimal for two stacked rhombic zigzag graphene nanofragments in our research. Thus this slipping is a spontaneous behavior induced by the spin polarization in the fully relaxed stacking process of these finite nanofragments. The conclusion may have an important significance in understanding the behaviors of free systems in nanoscale, which indicates spin polarization could present in small-sized adsorbed systems when the monomer possessing particular geometry and directly induces obvious mechanical effects. Besides this fundamental significance, the combination of spin-polarization and interlayer slippage may also be enlightening for applications, considering the spin-polarized graphene nanofragments with similar shapes and sizes are promised building blocks for spintronic devices^20^,^21^ while the slippage in bilayer graphene facilities the modulation of the electronic properties^19^,^22^,^23^.

This work, as a typical demonstration, pointed out the bridge between spin polarizations of nanoscale graphene structures and their complex structural conformations in intermolecular interactions. Meanwhile, as an example of intermolecular behaviors response to the monomeric spin polarization, we anticipate this finding to offer an inspiration for further explorations and designs.

## Methods

Simulations were implemented by first-principles calculations in Gaussian 09^24^. Optimizations employed the empirical dispersion-corrected density functional theory (DFT-D3) with hybrid generalized gradient-approximation (hybrid GGA) at PBE0-D3/6-31G(d) level^25^,^26^. The result was reproduced by the density functional theory (DFT) with hybrid meta-generalized gradient-approximation (hybrid meta-GGA) at the M06-2X/6-31G(d) level^27^. In order to find the energetically most favorable stacked structure, different initial conformations were fully optimized through both spin-polarized calculations and closed-shell calculations.

## Additional Information

**How to cite this article**: Lei, Y. *et al.* Slippage in stacking of graphene nanofragments induced by spin polarization. *Sci. Rep.* **5**, 10985; doi: 10.1038/srep10985 (2015).

## Supplementary Material

Supplementary Information
