# Supplementary material for: Slippage in stacking of graphene nanofragments induced by spin polarization
Source: Sci Rep. 2015 Jun 16;5:10985. doi: 10.1038/srep10985 (PMC4468519; doi:10.1038/srep10985)
Supplement: Supplementary Information [file srep10985-s1.doc]

**Supplementary Information**

Slippage in stacking of graphene nanofragments induced by spin polarization

Yanyu Lei^1,2#^, Wanrun Jiang^1,2#^, Xing Dai^1,2^, Ruixia Song^1,2^, Bo Wang^1,2^, Yang Gao^1,2^ & Zhigang Wang^1,2^*

^1^Institute of Atomic and Molecular Physics, Jilin University, Changchun 130012, People’s Republic of China, ^2^Jilin Provincial Key Laboratory of Applied Atomic and Molecular Spectroscopy (Jilin University), Changchun, 130012, People’s republic of China.

Correspondence and requests for materials should be addressed to Z.W. (wangzg@jlu.edu.cn).

**Supplementary Table S1 Atomic coordinates for the spin-**polarized conformation

| Atom | x(Å) | y(Å) | z(Å) |
| --- | --- | --- | --- |
| C | 2.12606253 | 3.66994841 | 0.01950981 |
| C | 0.74373712 | 3.69524640 | 0.01346595 |
| C | 0.00000000 | 4.92536240 | 0.00000000 |
| C | -2.11435370 | 3.71111215 | -0.01695583 |
| C | -1.35577697 | 4.93316163 | -0.01440885 |
| C | -3.49718475 | 3.69984557 | -0.03734331 |
| C | 6.38336842 | 1.17580618 | 0.01714509 |
| C | 4.96969780 | 1.21318672 | 0.03069588 |
| C | 4.25296303 | 2.42371877 | 0.02871746 |
| C | 2.13045744 | 1.21932051 | 0.02432658 |
| C | 2.85438618 | 2.45468196 | 0.02849221 |
| C | 0.71838735 | 1.22817100 | 0.01538296 |
| C | 0.01187768 | 2.46097660 | 0.00979900 |
| C | -2.11618454 | 1.24295244 | -0.01327970 |
| C | -1.39640030 | 2.46832034 | -0.00363260 |
| C | -3.52816416 | 1.24826851 | -0.02844372 |
| C | -4.23892821 | 2.49164586 | -0.04079177 |
| C | -6.36732402 | 1.27062574 | -0.07632232 |
| C | -5.63744754 | 2.47426103 | -0.06587864 |
| C | -7.78079623 | 1.24853578 | -0.11706887 |
| C | 6.37131011 | -1.23962168 | -0.00347756 |
| C | 7.06307055 | -0.03527319 | 0.00000000 |
| C | 4.95733073 | -1.26311064 | 0.00949449 |
| C | 4.24997643 | -0.02144585 | 0.02571336 |
| C | 2.11817332 | -1.24077691 | 0.00329380 |
| C | 2.83578994 | -0.01435292 | 0.02215975 |
| C | 0.70608558 | -1.23536723 | -0.00568224 |
| C | 0.00000000 | 0.00000000 | 0.00000000 |
| C | -2.12849013 | -1.22134461 | -0.03436851 |
| C | -1.41062435 | 0.00714224 | -0.01129397 |
| C | -3.54045128 | -1.21229763 | -0.04951166 |
| C | -4.24609002 | 0.02158321 | -0.0440546 |
| C | -6.37968820 | -1.20547856 | -0.09753134 |
| C | -5.66029977 | 0.02882423 | -0.06502383 |
| C | -7.79286553 | -1.16858145 | -0.13777012 |
| C | -8.47248545 | 0.04358325 | -0.14864732 |
| C | 4.22854269 | -2.46621282 | -0.01305653 |
| C | 2.08929989 | -3.69079546 | -0.04345961 |
| C | 2.82972738 | -2.48320004 | -0.01372145 |
| C | 0.70679373 | -3.70217721 | -0.04987202 |
| C | -0.01270186 | -2.46077798 | -0.03230066 |
| C | -2.15130985 | -3.68897675 | -0.08026834 |
| C | -1.42097952 | -2.45382517 | -0.04574191 |
| C | -3.53395518 | -3.66355209 | -0.10034816 |
| C | -4.26359728 | -2.44812054 | -0.08307985 |
| C | -5.66186834 | -2.41634373 | -0.10775339 |
| C | -0.04918924 | -4.92439389 | -0.08431935 |
| C | -1.40497590 | -4.91840595 | -0.09869731 |
| H | 6.93292658 | 2.11292209 | 0.00205695 |
| H | 4.80300813 | 3.36170785 | 0.01519821 |
| H | 2.67714857 | 4.60756994 | 0.00625862 |
| H | 0.55703896 | 5.85908158 | -0.00316892 |
| H | -1.90109045 | 5.87423202 | -0.02412857 |
| H | -4.03898027 | 4.64380803 | -0.04771975 |
| H | -6.17873442 | 3.41826615 | -0.07756604 |
| H | -8.32114439 | 2.19181635 | -0.12644147 |
| H | -8.34260693 | -2.10611850 | -0.16324415 |
| H | -6.21255449 | -3.35455732 | -0.13553721 |
| H | -4.08515122 | -4.60174043 | -0.12680817 |
| H | -1.95965839 | -5.85367970 | -0.12444818 |
| H | 0.49849663 | -5.86343755 | -0.10354090 |
| H | 2.63099784 | -4.63350376 | -0.07290504 |
| H | 4.76921514 | -3.40926987 | -0.04263137 |
| H | 6.91151879 | -2.18177426 | -0.0344678 |
| H | -9.55887668 | 0.04927633 | -0.18000496 |
| H | 8.14866869 | -0.04036591 | -0.03882968 |
| C | 4.49731950 | 3.68760287 | -3.30469434 |
| C | 3.11467001 | 3.71311867 | -3.32478694 |
| C | 2.36841345 | 4.94259107 | -3.30630927 |
| C | 0.25656735 | 3.72649971 | -3.35525019 |
| C | 1.01262590 | 4.94866862 | -3.32075780 |
| C | -1.12594249 | 3.71520935 | -3.36170563 |
| C | 8.75608480 | 1.19236408 | -3.26787201 |
| C | 7.34290529 | 1.22935500 | -3.30796971 |
| C | 6.62516422 | 2.44026638 | -3.29758432 |
| C | 4.50365709 | 1.23635478 | -3.35571455 |
| C | 5.22688559 | 2.47213212 | -3.32212828 |
| C | 3.09169580 | 1.24549092 | -3.37075920 |
| C | 2.38426247 | 2.47801570 | -3.35933809 |
| C | 0.25711357 | 1.25969331 | -3.39941285 |
| C | 0.97598238 | 2.48505650 | -3.37277724 |
| C | -1.15497494 | 1.26519330 | -3.40842422 |
| C | -1.86644692 | 2.50766243 | -3.39145747 |
| C | -3.99413269 | 1.28771315 | -3.41475407 |
| C | -3.26526410 | 2.49076583 | -3.39219282 |
| C | -5.40811269 | 1.26431793 | -3.40186769 |
| C | 8.74385342 | -1.22475370 | -3.28853006 |
| C | 9.43562697 | -0.01984579 | -3.25704650 |
| C | 7.33037448 | -1.24675179 | -3.32914005 |
| C | 6.62343185 | -0.00490298 | -3.34040541 |
| C | 4.49121225 | -1.22420880 | -3.37675387 |
| C | 5.20921968 | 0.00243102 | -3.36125267 |
| C | 3.07922781 | -1.21880350 | -3.39182488 |
| C | 2.37374740 | 0.01705121 | -3.39380145 |
| C | 0.24465064 | -1.20383818 | -3.42047013 |
| C | 0.96312105 | 0.02428220 | -3.40506416 |
| C | -1.16742063 | -1.19489942 | -3.42944402 |
| C | -1.87267486 | 0.03881794 | -3.42731518 |
| C | -4.00666043 | -1.18858588 | -3.43590035 |
| C | -3.28686182 | 0.04600124 | -3.43091020 |
| C | -5.42033214 | -1.15111313 | -3.42249056 |
| C | -6.09995194 | 0.06001438 | -3.40540908 |
| C | 6.60041731 | -2.45033961 | -3.33947514 |
| C | 4.46006890 | -3.67578657 | -3.36779273 |
| C | 5.20189462 | -2.46763354 | -3.36443090 |
| C | 3.07723619 | -3.68696287 | -3.38809462 |
| C | 2.35936149 | -2.44412238 | -3.40142664 |
| C | 0.21914502 | -3.67090876 | -3.41844012 |
| C | 0.95108408 | -2.43668780 | -3.41483936 |
| C | -1.16317690 | -3.64552198 | -3.42457722 |
| C | -1.89142765 | -2.43021419 | -3.43364332 |
| C | -3.29000018 | -2.39916028 | -3.43395484 |
| C | 2.31857761 | -4.90896349 | -3.39057715 |
| C | 0.96279992 | -4.90107375 | -3.40492669 |
| H | 9.30588918 | 2.12986628 | -3.24245202 |
| H | 7.17591382 | 3.37844493 | -3.26983345 |
| H | 5.04857638 | 4.62575960 | -3.27831528 |
| H | 2.92316089 | 5.87782751 | -3.28056133 |
| H | 0.46499927 | 5.88774701 | -3.30153886 |
| H | -1.66757980 | 4.65795270 | -3.33230224 |
| H | -3.80587584 | 3.43385786 | -3.36265098 |
| H | -5.94825704 | 2.20650749 | -3.37091387 |
| H | -5.96998659 | -2.08817787 | -3.40759807 |
| H | -3.84012776 | -3.33710150 | -3.42048790 |
| H | -1.71432724 | -4.58310307 | -3.41122345 |
| H | 0.40569963 | -5.83475506 | -3.40170692 |
| H | 2.86382970 | -5.85006851 | -3.38086464 |
| H | 5.00180459 | -4.61978342 | -3.35745288 |
| H | 7.14164466 | -3.39438007 | -3.32783040 |
| H | 9.28414024 | -2.16807069 | -3.27918985 |
| H | -7.18555323 | 0.06517624 | -3.36671131 |
| H | 10.52202036 | -0.02561096 | -3.22579652 |

**Supplementary Table S2 Atomic coordinates for the closed-shell conformation**

| Atom | x(Å) | y(Å) | z(Å) |
| --- | --- | --- | --- |
| C | 2.07719843 | -3.70190519 | 0.03285749 |
| C | 0.70472136 | -3.71161490 | 0.03095784 |
| C | -0.05737448 | -4.93598947 | 0.05031649 |
| C | -2.15746953 | -3.69519082 | 0.03990030 |
| C | -1.40920298 | -4.92845842 | 0.05096937 |
| C | -3.53017111 | -3.66966083 | 0.04993351 |
| C | 6.35622801 | -1.24415761 | 0.00421721 |
| C | 4.94919020 | -1.26598275 | -0.00422456 |
| C | 4.21313032 | -2.47368376 | 0.01360363 |
| C | 2.11122545 | -1.24226023 | -0.00081887 |
| C | 2.82738621 | -2.48868727 | 0.01215460 |
| C | 0.70446925 | -1.23609134 | 0.00145509 |
| C | -0.02121759 | -2.46430576 | 0.01805298 |
| C | -2.12935316 | -1.21978900 | 0.01932042 |
| C | -1.41752409 | -2.45639608 | 0.02177674 |
| C | -3.53622251 | -1.20970514 | 0.02987335 |
| C | -4.26653629 | -2.44787218 | 0.04924401 |
| C | -6.37413828 | -1.20081012 | 0.08149709 |
| C | -5.65241340 | -2.41701561 | 0.07193405 |
| C | -7.78129487 | -1.16293113 | 0.12035021 |
| C | 6.37022796 | 1.17016252 | -0.00809723 |
| C | 7.05000570 | -0.04097161 | 0.00000000 |
| C | 4.96354890 | 1.20824669 | -0.01681469 |
| C | 4.24313019 | -0.02475240 | -0.01505768 |
| C | 2.12551966 | 1.21754956 | -0.01332783 |
| C | 2.82924153 | -0.01652371 | -0.01390093 |
| C | 0.71878907 | 1.22776714 | -0.01110902 |
| C | 0.00000000 | 0.00000000 | 0.00000000 |
| C | -2.11502912 | 1.24461197 | 0.00666022 |
| C | -1.41075345 | 0.00822515 | 0.00357620 |
| C | -3.52191848 | 1.25099846 | 0.01714331 |
| C | -4.24003021 | 0.02483614 | 0.03379861 |
| C | -6.35974530 | 1.27563973 | 0.06862817 |
| C | -5.65434284 | 0.03318396 | 0.05769994 |
| C | -7.76723717 | 1.25454154 | 0.10780282 |
| C | -8.45931008 | 0.04989104 | 0.13408057 |
| C | 4.24157486 | 2.42454167 | -0.01128887 |
| C | 2.12007289 | 3.67765140 | -0.00463570 |
| C | 2.85610947 | 2.45564174 | -0.01296838 |
| C | 0.74781047 | 3.70329715 | -0.00679900 |
| C | 0.00742772 | 2.46444614 | -0.00708470 |
| C | -2.11437233 | 3.72025160 | 0.00183600 |
| C | -1.38887274 | 2.47281329 | -0.00346040 |
| C | -3.48727105 | 3.71079129 | 0.01179806 |
| C | -4.23779152 | 2.49770512 | 0.02362092 |
| C | -5.62392564 | 2.48320668 | 0.04647777 |
| C | 0.00000000 | 4.93658809 | 0.00000000 |
| C | -1.35182047 | 4.94478593 | 0.00042593 |
| H | 6.89704300 | -2.18660897 | 0.02871782 |
| H | 4.75597446 | -3.41610741 | 0.03716464 |
| H | 2.62018386 | -4.64440698 | 0.05579064 |
| H | 0.48959697 | -5.87543030 | 0.06772498 |
| H | -1.96605498 | -5.86260463 | 0.06503191 |
| H | -4.08390642 | -4.60662569 | 0.06331982 |
| H | -6.20606713 | -3.35380384 | 0.08803005 |
| H | -8.33328402 | -2.09947065 | 0.13853238 |
| H | -8.30828955 | 2.19757824 | 0.11628292 |
| H | -6.16664887 | 3.42648560 | 0.05284828 |
| H | -4.03007566 | 4.65421920 | 0.01538376 |
| H | -1.89777649 | 5.88543839 | 0.00485347 |
| H | 0.55786855 | 5.86973005 | 0.00800206 |
| H | 2.67398420 | 4.61395783 | 0.00877850 |
| H | 4.79534116 | 3.36078100 | 0.00270453 |
| H | 6.92195134 | 2.10647606 | 0.00680390 |
| H | -9.54589785 | 0.05635823 | 0.16274112 |
| H | 8.13552807 | -0.04713338 | 0.02735180 |
| C | 3.62579637 | -3.69575337 | 3.48658715 |
| C | 2.25289241 | -3.70444982 | 3.49708634 |
| C | 1.48966445 | -4.92855816 | 3.49885097 |
| C | -0.60925917 | -3.68589793 | 3.50621566 |
| C | 0.13784753 | -4.91960286 | 3.49955689 |
| C | -1.98151007 | -3.65949656 | 3.50399079 |
| C | 7.90708947 | -1.24189787 | 3.38884021 |
| C | 6.49959397 | -1.26221989 | 3.42861629 |
| C | 5.76312010 | -2.46937177 | 3.45096962 |
| C | 3.66182641 | -1.23597615 | 3.48121003 |
| C | 4.37698174 | -2.48309086 | 3.47445895 |
| C | 2.25494654 | -1.22880460 | 3.49209389 |
| C | 1.52810722 | -2.45660346 | 3.50245923 |
| C | -0.57884909 | -1.21038742 | 3.51013907 |
| C | 0.13181324 | -2.44745728 | 3.50628335 |
| C | -1.98557157 | -1.19939734 | 3.51226682 |
| C | -2.71685117 | -2.43707770 | 3.51202098 |
| C | -4.82359334 | -1.18853174 | 3.51493243 |
| C | -4.10230124 | -2.40522579 | 3.50994002 |
| C | -6.23024855 | -1.14967709 | 3.50537254 |
| C | 7.92251311 | 1.17556329 | 3.37664591 |
| C | 8.59983339 | -0.03764197 | 3.36247025 |
| C | 6.51540158 | 1.21422883 | 3.41603631 |
| C | 5.79491006 | -0.01935631 | 3.43996159 |
| C | 3.67751174 | 1.22472125 | 3.46852324 |
| C | 4.38061768 | -0.01021293 | 3.46437394 |
| C | 2.27065427 | 1.23558919 | 3.47929549 |
| C | 1.55136757 | 0.00797616 | 3.49521384 |
| C | -0.56315270 | 1.25346183 | 3.49722539 |
| C | 0.14062191 | 0.01697964 | 3.49885649 |
| C | -1.96990151 | 1.26040284 | 3.49933397 |
| C | -2.68860046 | 0.03506298 | 3.51250622 |
| C | -4.80782916 | 1.28568924 | 3.50198314 |
| C | -4.10247952 | 0.04406059 | 3.51327099 |
| C | -6.21486437 | 1.26463316 | 3.49278346 |
| C | -6.90932754 | 0.06184059 | 3.49666111 |
| C | 5.79436749 | 2.43084002 | 3.42596488 |
| C | 3.67284384 | 3.68467564 | 3.44849243 |
| C | 4.40852096 | 2.46247340 | 3.44903144 |
| C | 2.30016481 | 3.71097169 | 3.45855930 |
| C | 1.55952022 | 2.47259078 | 3.47678862 |
| C | -0.56201382 | 3.72898400 | 3.46735721 |
| C | 0.16322214 | 2.48127225 | 3.48047656 |
| C | -1.93448803 | 3.72003340 | 3.46528584 |
| C | -2.68535667 | 2.50723087 | 3.48603282 |
| C | -4.07109933 | 2.49298364 | 3.48425139 |
| C | 1.55258843 | 4.94465764 | 3.44740401 |
| C | 0.20076839 | 4.95293591 | 3.44791282 |
| H | 8.44761190 | -2.18523606 | 3.38003374 |
| H | 6.30530982 | -3.41295323 | 3.44425539 |
| H | 4.16807772 | -4.63948116 | 3.48279708 |
| H | 2.03509732 | -5.86951134 | 3.49444188 |
| H | -0.42054400 | -5.85243227 | 3.49181266 |
| H | -2.53594776 | -4.59549244 | 3.49063843 |
| H | -4.65657979 | -3.34116085 | 3.49597157 |
| H | -6.78249353 | -2.08567445 | 3.49027670 |
| H | -6.75514846 | 2.20738131 | 3.46790177 |
| H | -4.61342451 | 3.43570206 | 3.46048494 |
| H | -2.47695277 | 4.66282996 | 3.44215040 |
| H | -0.34569082 | 5.89267253 | 3.43029529 |
| H | 2.10996465 | 5.87849133 | 3.43328145 |
| H | 4.22710647 | 4.62132789 | 3.43503333 |
| H | 6.34854375 | 3.36731404 | 3.40967526 |
| H | 8.47502261 | 2.11179199 | 3.35834317 |
| H | -7.99482028 | 0.06860483 | 3.46847035 |
| H | 9.68640487 | -0.04472331 | 3.33338227 |

**Supplementary Table S3 Comparison of energy difference between conformations with other**

**spin-coupling patterns and the AFM-AFM pattern**.

| Spin-coupling pattern | ΔE^a^ (eV) |
| --- | --- |
| AFM-AFM  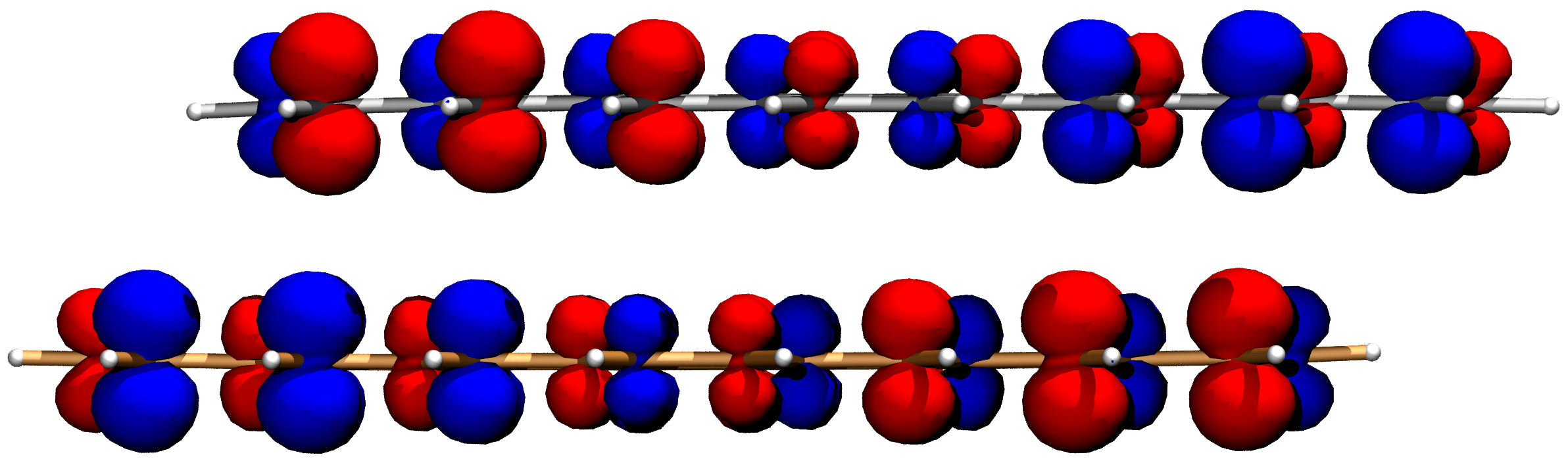 | 0 |
| AFM-FM  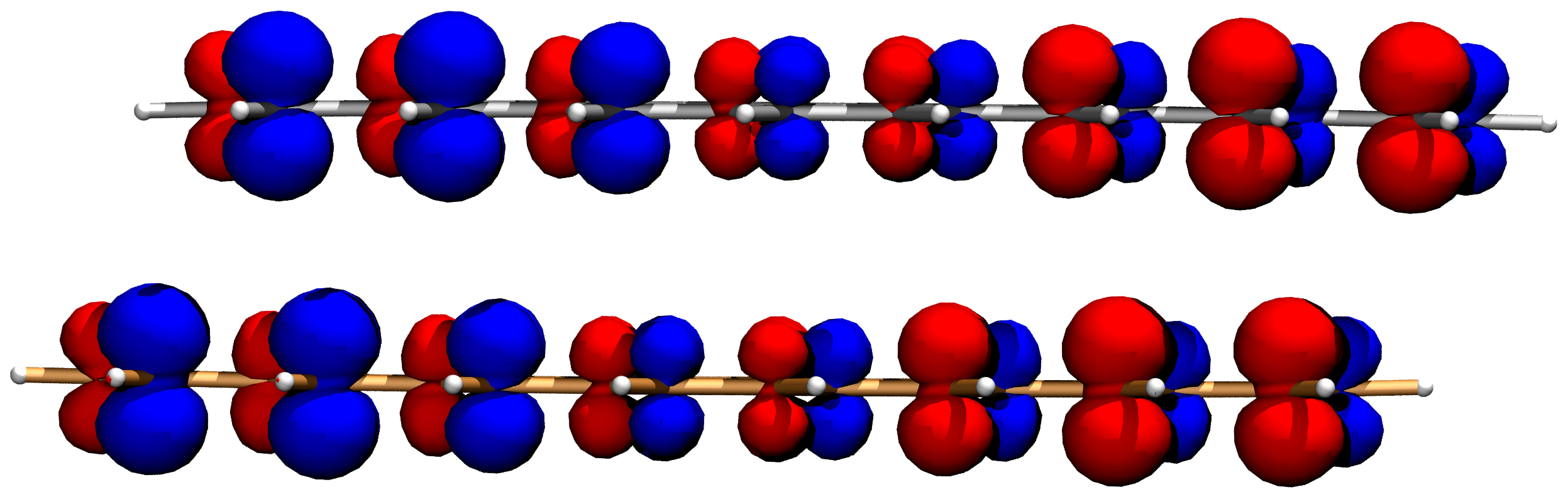 | 0.002 (0.05) |
| FM-AFM  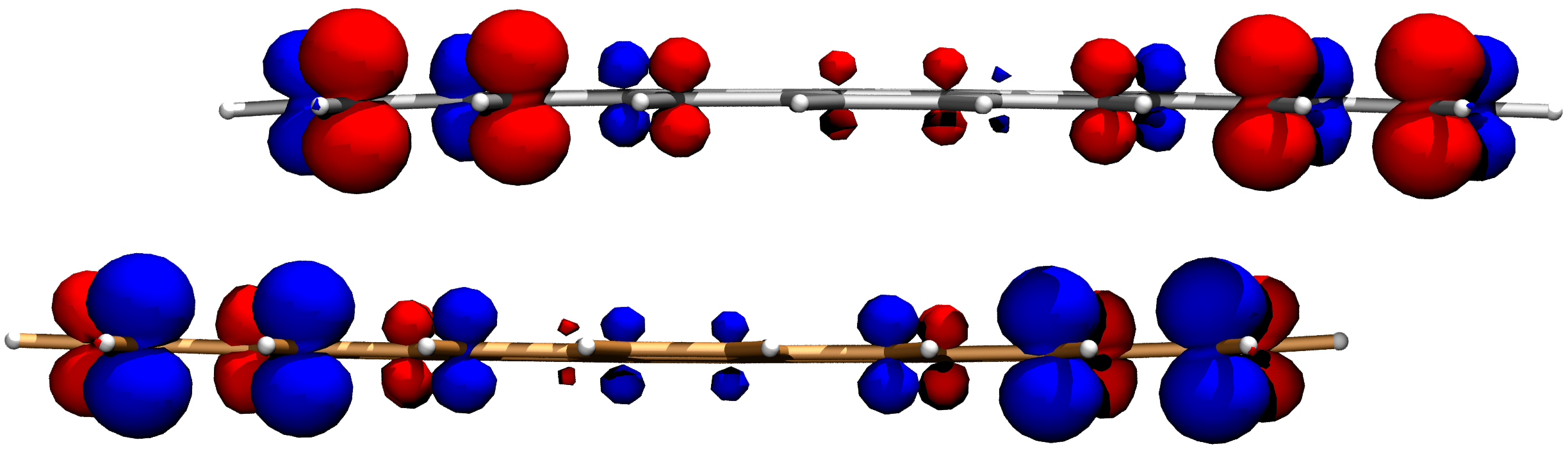 | 0.48 |
| FM-FM  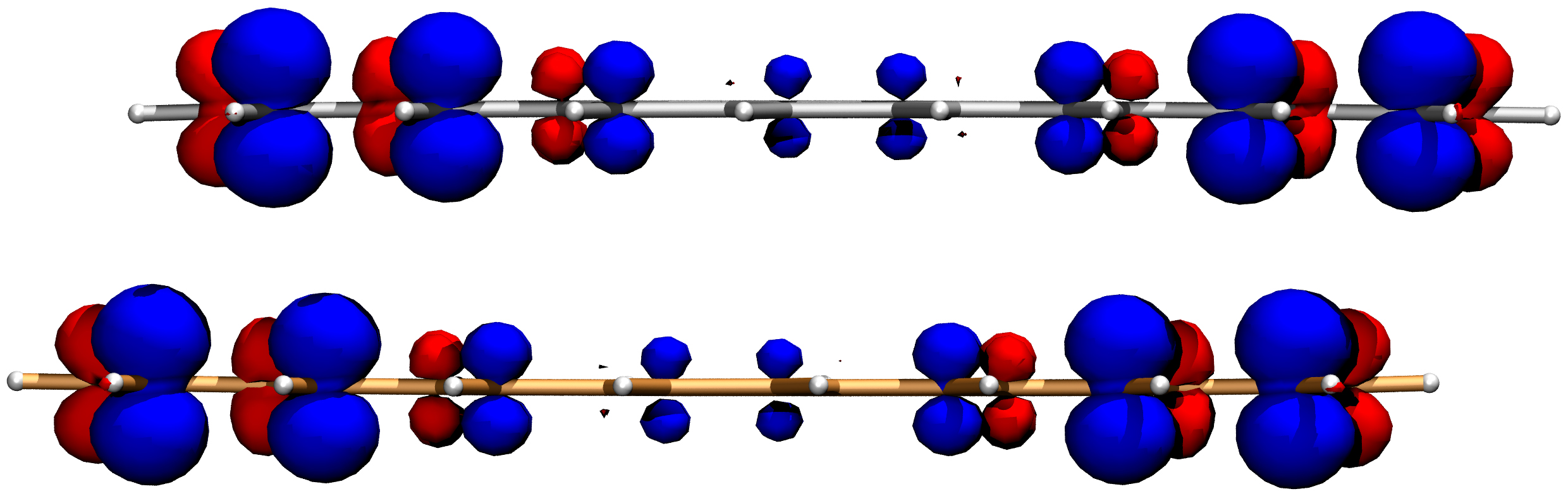 | 0.53 |
| AFM&FM  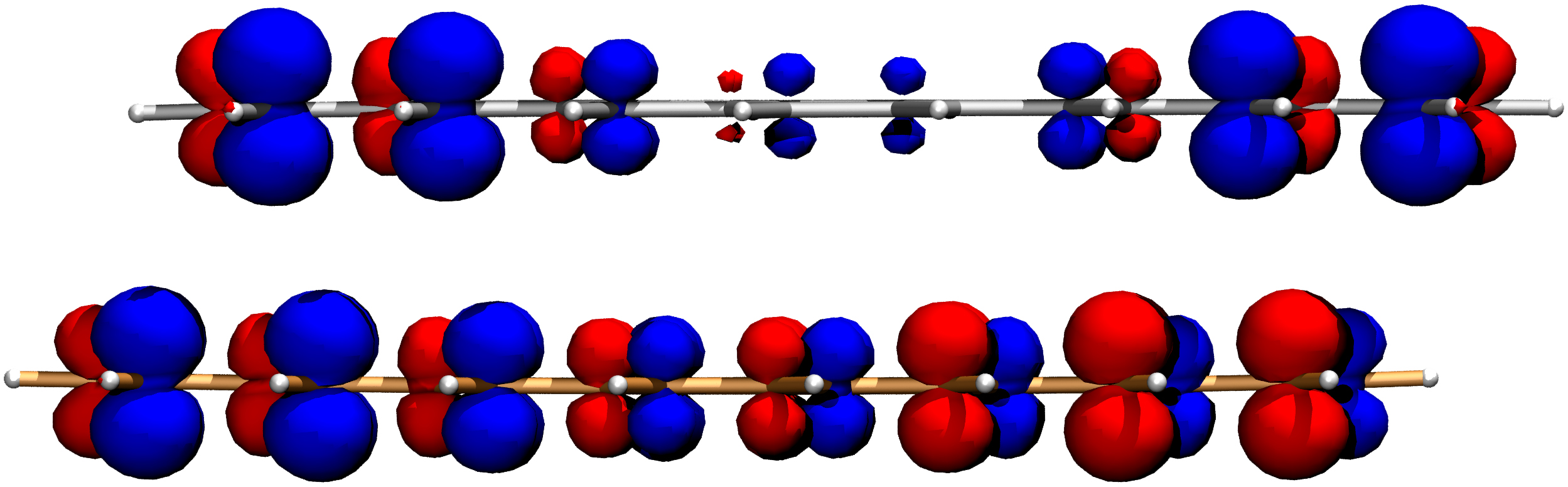 | 0.26 |

^a^ The values without parentheses is calculated at PBE0-D3/6-31G(d) level. The value in the parentheses is calculated at M06-2X/6-31G(d) level.

Four usually discussed coupling patterns are considered, namely the AFM-AFM (antiferromagnetic and antiferromagnetic couplings for the intra-layer and inter-layer spin arrangements, respectively), FM-AFM (ferromagnetic and antiferromagnetic couplings for the intra-layer and inter-layer spin arrangements, respectively), FM-AFM and FM-FM couplings. Besides, another case found in our calculation is also given, where one fragment shows antiferromagnetic intra-layer coupling and the other fragment shows ferromagnetic intra-layer coupling (noted as AFM&FM). The conformation possessing the AFM-AFM pattern shows the lowest total energy. Energy differences of other cases relative to this AFM-AFM one are given in Supplementary Table S3. The results are calculated at PBE0-D3/6-31G(d) level. Optimizations at M06-2X/6-31G(d) were carried out to further determine the optimal conformation between the AFM-FM one and the AFM-AFM one. The energy preference for AFM-AFM coupling and the small energy difference between AFM-AFM and AFM-FM coupling patterns are in consistent with the study on bilayer zigzag graphene nanoribbons in reference 18.

**Supplementary Table S4 Comparison of energy differences between other states and AFM**

**states of the single rhombic graphene nanofragment in different sizes.**

| Size | ΔE^a^ (eV) | | | | | |
| --- | --- | --- | --- | --- | --- | --- |
|  | AFM | Closed-shell | FM | | | |
|  |  |  | 3 | 5 | 7 | 9 |
| 4 | 0 | 0.21 | **0.26** | 1.33 | - | - |
| 5 | 0 | 0.71 | **0.27** | 0.50 | - | - |
| 6 | 0 | 1.34 | 0.62 | **0.26** | 1.02 | - |
| 7 | 0 | 1.99 | 1.14 | 0.40 | **0.37** | 1.32 |
| 8 | 0 | 2.77 | 2.03 | 0.79 | **0.31** | 0.59 |

^a^ The numbers in bold are the energy differences between the FM state with the lowest total energy and the AFM state.

The size of single rhombic graphene nanofragments (GNFs) is noted by the number of zigzag edge atoms on each edge of the fragment (for example, the size of that we used in the main text is 4). The size we compared is from 4 to 8. Ferromagnetic (FM) states under multiplicities of 3, 5, 7 and 9 are considered. The energy differences between other states and the antiferromagetic (AFM) ground state are shown in Supplementary Table S4. Calculations were implemented at PBE0-D3/6-31G(d) level. The results show, AFM configuration is optimal in all compared sizes. The conclusion that AFM states are optimal spin-polarized states for zigzag graphene structures is in consistent with those in reference 6-10.

**Supplementary Table S5 Related information about the size effect on the stacking system.**

| Size | Closed-shell structure | Displacement (Å) | Spin-polarized structure | Spin-coupling pattern |
| --- | --- | --- | --- | --- |
| 3-3 | AB | _ | _ | _ |
| 4-4 | AB | 1.23 | slipped^a^ | AFM-AFM |
| 5-5 | AA | 1.48 | close to AB | AFM-FM |

^a^ slipped structure refers to the structure has the interlayer displacement larger than that of the AB stacking.

With the same notation of the size used in Supplementary Table S4, the size of bilayer stacked system in the main text is noted as 4-4. Considering the computational efficiency, we carried out optimizations for stacked GNFs form 3-3 to 5-5 at PBE0-D3/3-21G level for a qualitative conclusion. Related information are shown in Supplementary Table S5. It’s shown that, the most stable structure in closed-shell state varies from AB stacking to AA stacking at size 5-5. Meanwhile, the geometric structure and spin-coupling pattern of the optimal spin-polarized conformation is also different between 5-5 system and 4-4 system. These changes mean the reference structure and the final conformation in the comparison is altered, where the relative slippage arises from. This made the displacement might not correspond to a simple trend when size increase in a small range. For size 3-3, the optimized conformation with the rhombic monomer possessing *S* = 0 spin-polarized states cannot be got and this result is verified by lager basis sets of 6-31G(d). This result is in consistent with reference 8 indicating a similar sized rectangular shape is the smallest graphene fragment which can possess spin polarization.

On the other hand, when size increases from 4-4, the optimal closed-shell conformation has higher energy than the most stable closed-shell conformation. And the stacking patterns of such two conformations are different. Meanwhile, shown by Supplementary Table S3, the stability of optimal AFM spin-polarized states of rhombic GNF relative to its other states wouldn’t decay as size increasing from 4 to 8. Combining these two discussions, it’s suggested that the mechanical effect induced by spin-polarization would occur when size increases in a small range.

**Supplementary Table S6 Energy differences between twisted conformations and the optimal slipped conformation.**

| No. | Twisted structure | Spin-coupling pattern | ΔE (eV) |
| --- | --- | --- | --- |
| 1 | 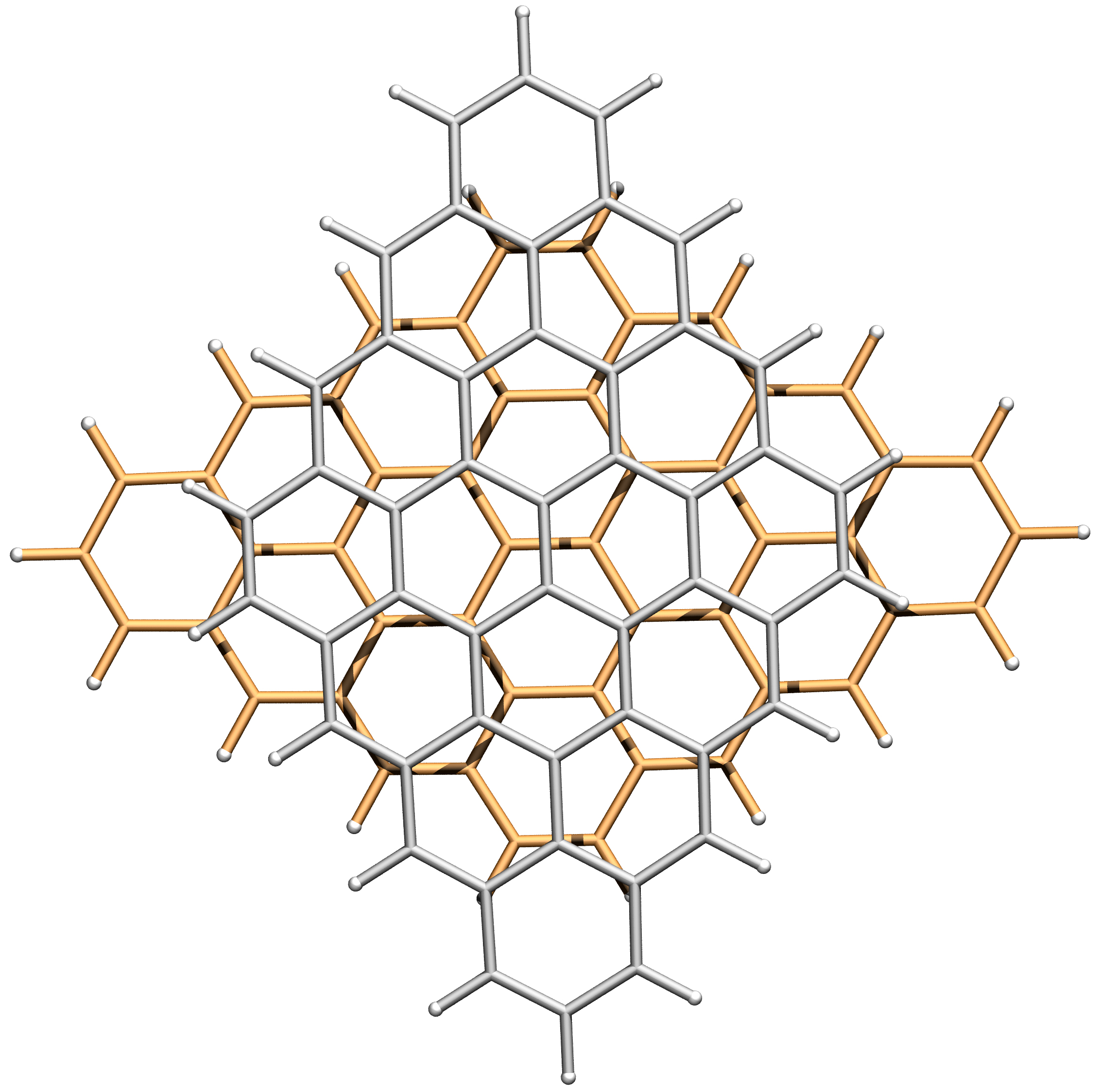 | Twisted AFM-AFM  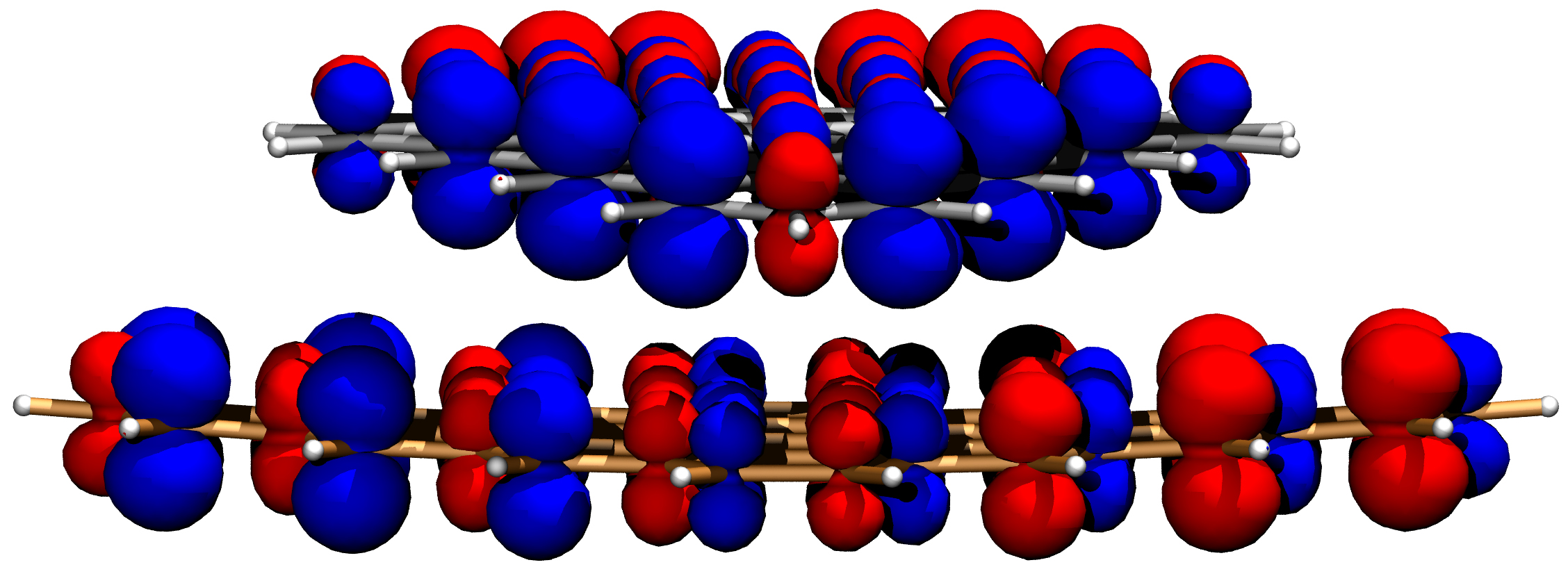 | 0.15 |
| 2 | 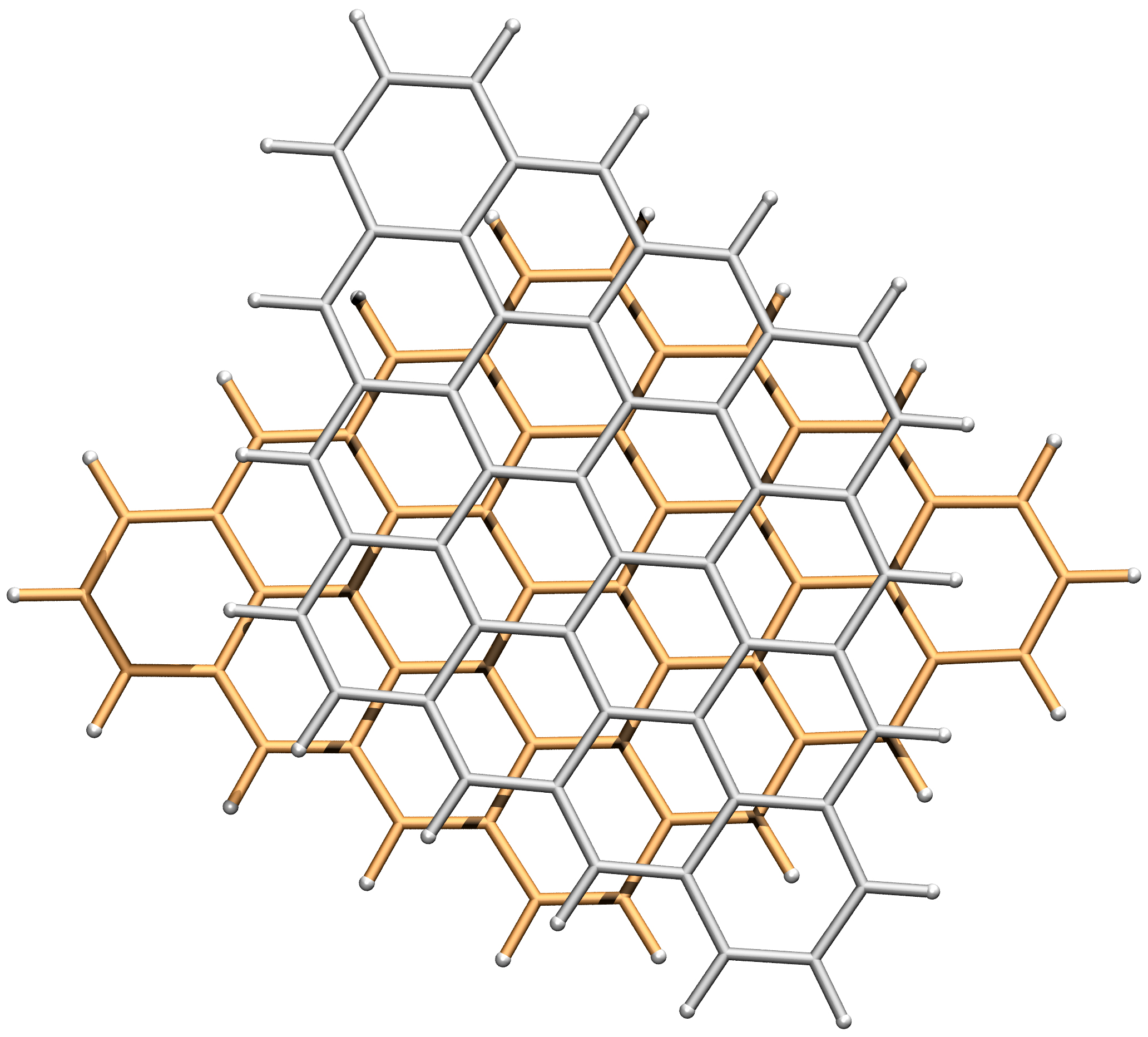 | Twisted AFM-AFM  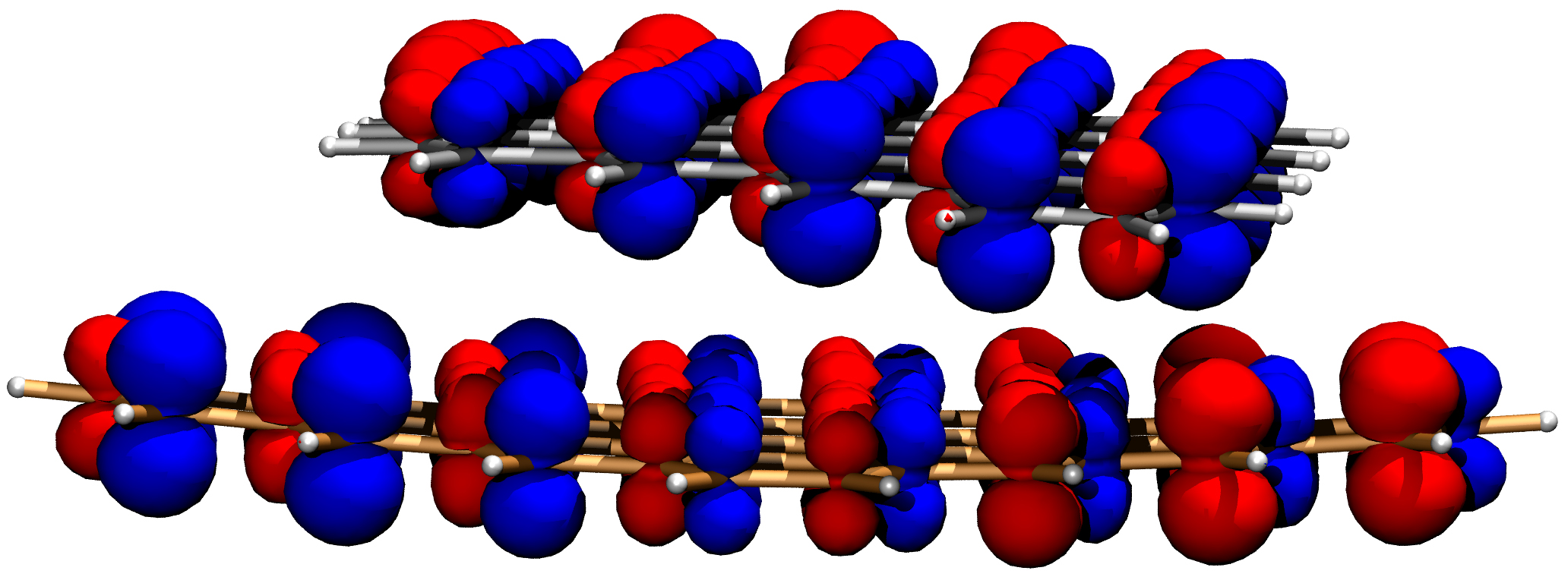 | 0.01 |
| 3 | 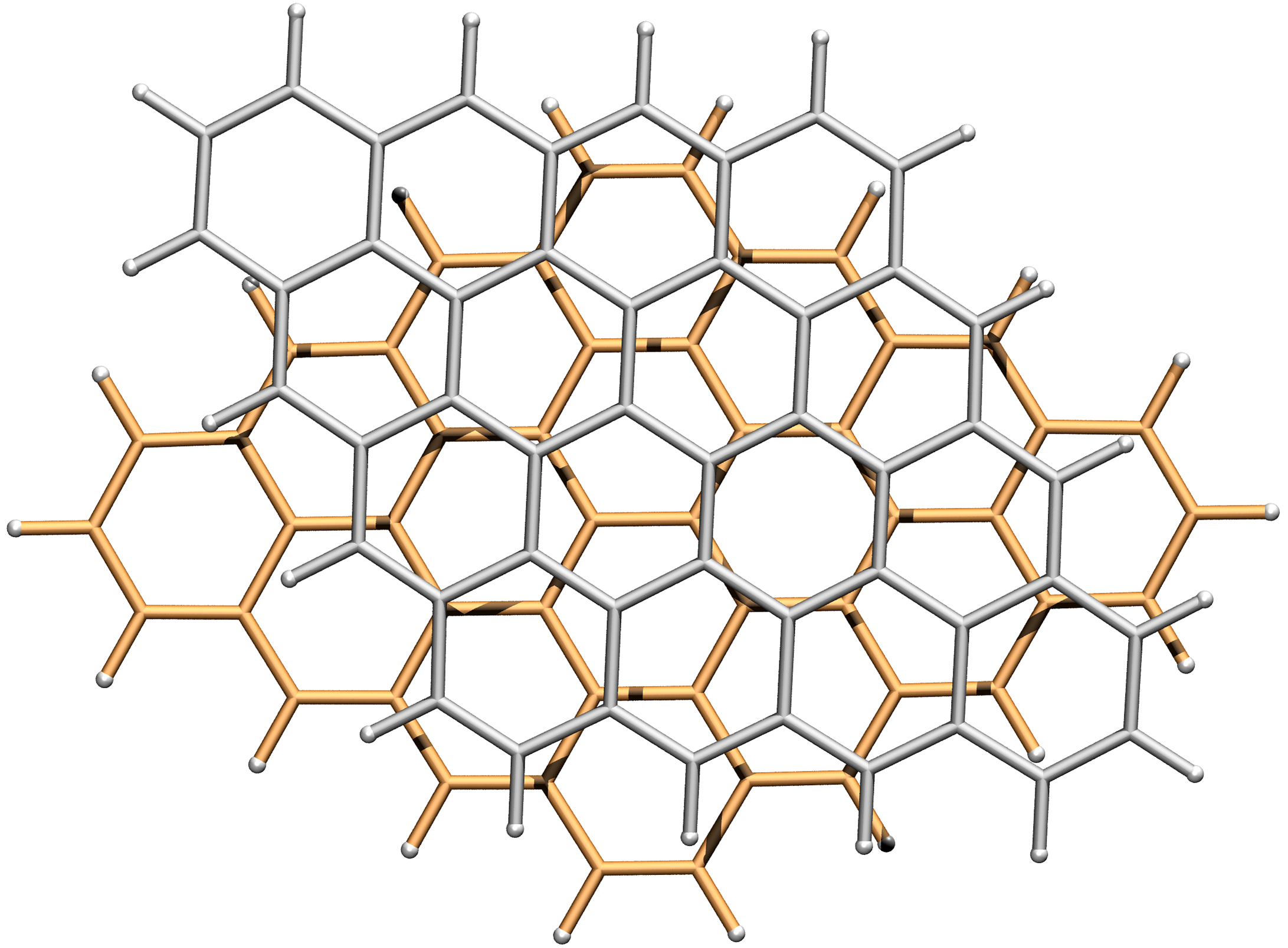 | Twisted AFM-AFM  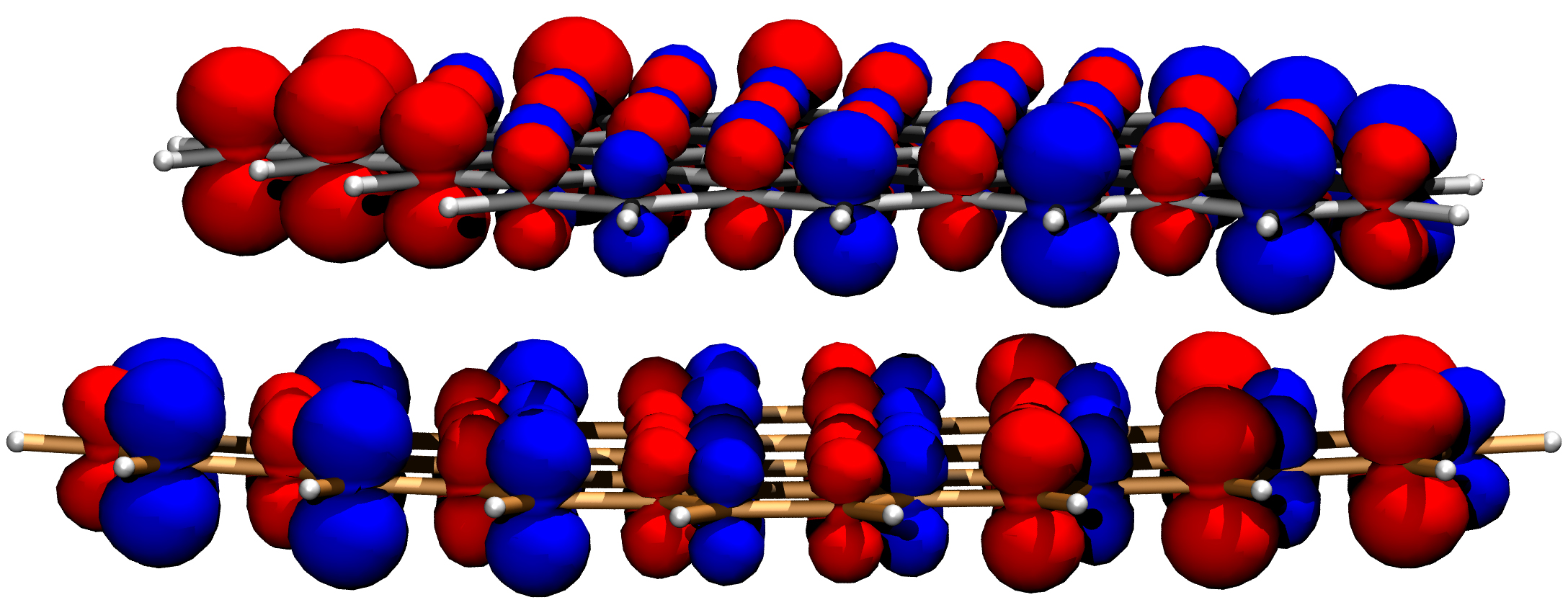 | 0.05 |
| 4 | 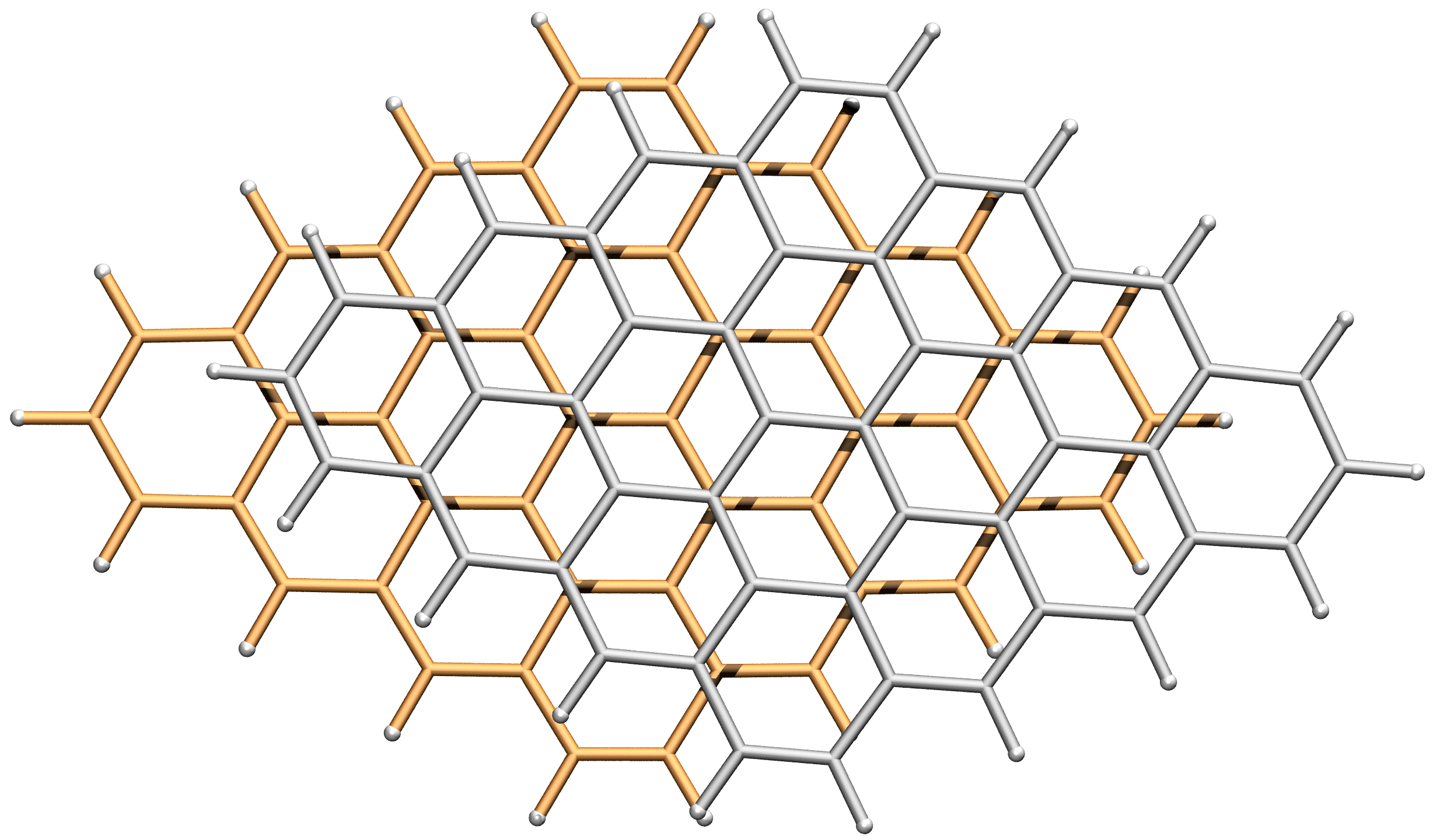 | Twisted FM-FM  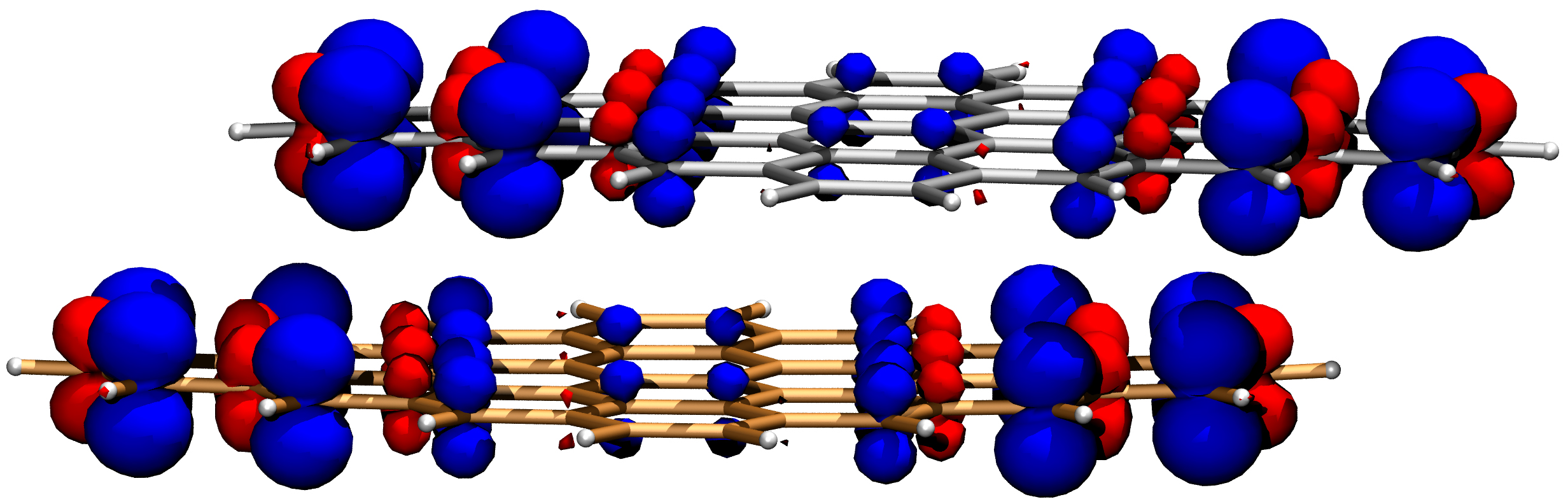 | 0.55 |
